# Adhesion factors and antimicrobial resistance of *Escherichia coli* strains associated with colibacillosis in piglets in Colombia

**DOI:** 10.14202/vetworld.2023.1231-1237

**Published:** 2023-06-05

**Authors:** Omar V. Pabón-Rodríguez, Karina López-López, Gloria A. Casas-Bedoya, José D. Mogollón-Galvis, Liliana Serna-Cock

**Affiliations:** 1 Departamento de Ciencia Animal, Facultad de Ciencias Agropecuarias, Universidad Nacional de Colombia, Palmira, Colombia; 2 Departamento de Ciencias Biológicas, Facultad de Ciencias Agropecuarias, Universidad Nacional de Colombia, Palmira, Colombia; 3 Departamento de Producción Animal, Facultad de Medicina Veterinaria y de Zootecnia, Universidad Nacional de Colombia, Bogotá, Colombia; 4 Departamento de Salud Animal, Facultad de Medicina Veterinaria y de Zootecnia, Universidad Nacional de Colombia, Bogotá, Colombia; 5 Departamento de Ingeniería, Facultad de Ingeniería y Administración, Universidad Nacional de Colombia, Palmira, Colombia

**Keywords:** adhesive fimbriae, piglet diarrhea, enteroaggregative heat-stable toxin 1, enterotoxigenic *Escherichia* *coli*, hemolytic capacity

## Abstract

**Background and Aims:**

The pathogenicity of *Escherichia coli* is determined by the presence of genes that mediate virulence factors such as adherence capacity and toxin production. This research aimed to identify the adhesion factors and antibiotic resistance capacity of *E. coli* strains associated with diarrhea in piglets in Colombia.

**Materials and Methods:**

Presumptive *E. coli* strains were isolated from the rectal swabs of piglets in swine farms between 4 and 40 days of age with evidence of diarrhea. Presumptive *E. coli* strains were tested for antibiotic resistance. The hemolytic capacity of presumptive *E. coli* strains was measured and molecularly identified. Strains confirmed as hemolytic *E. coli* was evaluated for the presence of five adhesion factors (F4, F5, F6, F18, and F41) and resistance to 11 antibiotics.

**Results:**

Fifty-two putative *E. coli* strains were isolated, six of which showed a hemolytic capacity. The hemolytic strains were molecularly identified as *E. coli*. Adhesive fimbriae were found in five of six β-hemolytic *E. coli* isolates. Combinations of the adhesion factors F6–F18 and F6–F41 were linked to antibiotic resistance capacity.

**Conclusion:**

The phenomenon of *E. coli* strains resistant to multiple antibiotics on pig farms represents a constant risk factor for public health and pig production.

## Introduction

*Escherichia coli* is divided into non-pathogenic and pathogenic types, and it is part of the microbial intestinal microbiomes of endothermic animals. Non-pathogenic *E. coli* is a commensal organism in the intestinal lumen, whereas pathogenic *E. coli* is a causative agent of intestinal and extraintestinal diseases in humans and animals. *Escherichia coli* strains are also classified into different “pathotypes” based on the presence of virulence factors [1]. These virulence factors are molecules produced by microorganisms that cause interactions with the host. There are several pathotypes of *E. coli*: Enterotoxigenic *E. coli* (ETEC), necrotoxigenic *E. coli*, enteropathogenic *E. coli*, and enteroaggregative *E. coli* [2, 3]. The main virulence factors of *E. coli* pathotypes are adhesins with hair-like appendages (fimbriae or pili) and enterotoxins (peptides or proteins) [4]. The ETEC pathotype of *E. coli* is characterized by the presence of fimbriae called adhesion factors (F4, F5, F6, F17, and F18) and toxins that are thermolabile (LT) or thermostable (STa, STb, and enteroaggregative heat-stable toxin 1 [EAST1]) [5]. The detection of any combination of fimbriae and toxins could be classified as a specific pathotype, as suggested by Gomes *et al*. [6] and Luppi [2]. DNA-based molecular detection methods (polymerase chain reaction [PCR] and DNA hybridization) are generally used to detect *E. coli* virulence factors [7], and real-time PCR is used to detect ETEC [8]. However, routine *E. coli* detection methods are needed before measuring the gene expression and finding new virulence factors [9]. The prevalence of ETEC virulence factors has been reported in several countries; for example, the prevalence of these strains was 44% in Sao Paulo, Brazil [10], 17.6% in Southern Germany [11], and 43% in Mexico [12].

Diarrheal disease caused by *E. coli* in intensive swine farms is one of the most common diseases in neonatal and weaned piglets, and it has a significant economic impact on pig farming due to its association with high morbidity and mortality rates [13]. Enterotoxigenic *E. coli* is the most prevalent cause of severe and watery diarrhea in lactating and nursery pigs [2, 3]. Post-weaning diarrhea is usually related to F4+ (K88) and F18+ *E. coli* infections [5]. Treatment of this disease requires intensive antibiotic use, leading to antibiotic resistance in *E. coli* strains. Antibiotic-resistant *E. coli* is one of the most relevant problems in pig production [14–16]. In swine production, antimicrobial resistance results from inadequate management practices, poor hygiene, increased therapeutic use of various antimicrobial agents, and the incorporation of antibiotics as growth promoters [17, 18]. The prevalence of antimicrobial resistance is increasing dangerously, representing a global public health concern.

In Colombia, to control piglet diarrhea, each pig farm can have its own treatment approach for the selection of antibiotics, which might result in the emergence of *E. coli* strains with resistance to most commercially available antibiotics [19]. In intensive swine production systems, antibiotic resistance in *E. coli* varies between countries and regions within the same country, which is a concern for public health systems [20]. In Valle del Cauca, which is the largest pig-producing region in Colombia, there are no scientific reports of pathogenic *E. coli* strains associated with diarrhea in piglets.

This study investigated the presence of virulence factors and antimicrobial resistance in *E. coli* isolates from pigs with diarrhea in the largest Colombian swine-producing region.

## Materials and Methods

### Ethical approval

The study was approved by the Ethical Committee of the Universidad Nacional de Colombia, Palmira campus (Act N°. 02 of 2019).

### Study period and location

The study was conducted from July 2019 to April 2023, from the sampling of the piglets, until the completion of the tests in the Bioconversions and Molecular Biology Laboratories of the National University of Colombia, Palmira Campus. The study was conducted in the Department of Valle del Cauca, Colombia.

### Piglet selection

Seventy-seven piglets were selected at random from six intensive swine farms in Valle del Cauca, Colombia. The criterion for this selection was based on records of the presence of diarrhea in piglets. Samples were taken in the towns of Yumbo (3° 34’ 56’’ N, 76° 29’ 29’’ W), Palmira (3° 31’ 1’’ N. 76° 18’ 0’’ W). Information about rectal swab samples collected from piglets during the suckling or preweaning phases is presented in Table-1

**Table-1:** Description of sampling sites (Valle del Cauca, Colombia), number of samples per site and age of animals sampled.

Location	Number of samples	Age of piglets
Corregimiento Buitrera Municipality of Yumbo	6	21 days
Corregimiento de Tablones Municipality of Palmira	8	28 days
Potrerillo Municipality of Palmira	9	4–25 days
Corregimiento El Bolo Municipality of Palmira	10	21 days
Corregimiento Villa Gorgona Municipality of Candelaria	11	13–20 days
La Zapata Municipality of Palmira	8	8–40 days
Total samples	52	

On each farm, piglets aged 4–40 days, which represented the lactating, or nursery phase, were chosen. The affected piglets were passing loose or watery feces, exhibiting decreased feed intake, dehydration, and lethargy. Rectal swabs were collected in sterile plastic tubes containing 5 mL of sterile nutrient broth. The tubes were stored at 4°C in portable coolers with refrigerated gels and transported to the laboratory for processing.

### Isolation of presumptive strains of *E. coli*

To isolate β-hemolytic, adherent, and antibiotic-resistant *E. coli* strains from each swab sample, successive cultures using MacConkey agar and Chromocult medium (Merck, Germany) were performed until pure strains were obtained. Colonies with dark blue to violet coloration were considered presumptive *E. coli* and cryopreserved in glycerol (glycerol: inoculated soy broth = 1:1) at −60°C (deep freezer, New Brunswick, NJ, USA). The strains were collected with permission from the Ministry of Environment and Sustainable Development (Addendum N° 28 of the framework contract for access to genetic resources and their derived products N° 121 of January 22, 2016).

### Antibiotic resistance

Presumptive *E. coli* strains were tested for resistance to nine antibiotics as follows: Amikacin (AK, 30 μg), ampicillin (AMP, 10 μg), ciprofloxacin (CIP, 5 μg), doxycycline (DO, 30 μg), enrofloxacin (ENR, 5 μg), florfenicol (FFC, 30 μg), fosfomycin (FOS, 50 μg), gentamicin (CN, 10 μg), and norfloxacin (NOR, 10 μg). Presumptive *E. coli* strains with hemolytic capacity (as described in the next section) were also tested for resistance to apramycin (APR, 15 μg) and ceftiofur (EFT, 30 μg). The Kirby–Bauer (antibiogram) method was used according to the guidelines of the Clinical and Laboratory Standards Institute [21]. To determine the susceptibility of the strains to different antibiotics, the antibiogram strains were analyzed in triplicate.

### Hemolytic capacity (β-hemolysis)

The isolates were cultured on blood agar containing 5% sheep blood, and the presence of hemolysis was determined visually. Sterile-defibrinated lamb red blood (5% v/v) was used. Plates were incubated for 24 h at 37°C. Colonies with a translucent area around the colonies were identified as β-hemolytic strains [22]. The hemolytic activity was evaluated, and biochemical methods were used to further characterize single coliform colonies.

### Molecular identification of *E. coli* isolates

The presumptive β-hemolytic *E. coli* strains were confirmed by evaluating the 16S ribosomal gene using the universal oligonucleotide primers 27 F and 1492 R [23]. Genomic DNA of *E. coli* isolates was extracted by boiling with the following modifications. *Escherichia coli* were grown on MacConkey agar and incubated for 24 h at 37°C. A single bacterial colony was picked up with a microbiological loop and diluted in 50 μL of Milli-Q water in a sterile plastic tube. The sample was heated at 95°C for 5 min and centrifuged at 9464× *g* for 5 min, and the supernatant was recovered. Aliquots of 5 μL of supernatant were used in PCR.

PCR was performed in 25-μL volumes with the following components: 1× PCR buffer, 2 mM dNTP mix, 10 μM of each primer (primer 1492R: 5′-GGY-TAC-CTT-GTT-ACG-ACG-AC-TT-3′; primer 27F: 5′-AGA-GTT-TGA-TCM-TGG-CTC-AG-3′), 2 U of Taq DNA polymerase, 12.6 μL of Ultrapure water, and 5 μL of each DNA sample [23]. The PCR conditions were as follows: initial denaturation for 5 min at 94°C; 30 cycles of 1 min at 95°C, 1 min at 55°C, 1 min at 72°C; and final incubation for 7 min at 72°C. The PCR products were visualized by electrophoresis on a 0.8% agarose gel, and the image was taken using a photo documentation kit (Nippon Genetics, Japan).

The amplified fragments were sequenced by the Sanger method at Macrogen (Seoul, South Korea). Sequencing reads were edited and assembled using CLC Main Workbench software (Qiagen^®^, Hilden, Germany). The obtained 16S ribosomal gene sequences were analyzed against sequences found in the 16S ribosomal RNA (Bacteria and Archaea) GenBank database using the BLASTn (https://blast.ncbi.nlm.nih.gov/Blast.cgi) program available through the National Center for Biotechnology (https://www.ncbi.nlm.nih.gov/). Finally, the nucleotide sequences of each *E. coli* strain detected in this study were deposited in the GenBank ribosomal RNA database (https://www.ncbi.nlm.nih.gov/genbank/).

### Adhesion factors

Multiplex PCR was used to detect genes encoding adhesins F4, F5, F6, F18, and F41 of *E. coli* β-hemolytic strains as previously described [24]. For this purpose, bacterial DNA extraction was performed using the GeneJET Genomic DNA Purification Kit N° K 0721 – 0722 (Thermo Scientific, Lithuania) following the manufacturer’s recommendations.

The strains were sent to an external diagnostic laboratory to detect these adhesion factors, and the bacterial DNA extracted in the previous step was examined using an AccuPower ETEC – Pili 5-Plex PCR kit (Bionneer, Korea).

### Enterotoxin analysis

The presence of LT, STa, and STb enterotoxins, and EAST1 was established in *E. coli* strains that exhibited β-hemolysis using the boiling method as described previously [25].

PCR was performed in a 25-μL volume containing the same components as previously mentioned, except for the use of the following primers: LT toxin (targeting the eltB gene), 5′-TTA-CGG-CGT-TAC-TAT-CCT-CTC-TA-3′ (F), and 5′-GGT-CTC-GGT-CAG-ATA-TGT-GAT-TC-3′ (R); STa toxin (targeting the estA gene), 5′-TCC-CCT-CTT-TTA-GTC-AGT-CAA-CTG-3′ (F), and 5′-GCA-CAG-GCA-GGA-TTA-CAA-CAA-AGT-3′ (R); STb toxin (targeting the estB gene), 5′-GCA-ATA-AGG-TTG-AGG-TGA-T-3′ (F), and 5′-GCC-TGC-AGT-GAG-AAA- GG AC-3′ (R); and EAST1 (targeting the astA gene), 5′-CCA-TCA-ACA-CAG-TAT-ATC-CGA-3′ (F), and 5′-GGT-CGC-GAG-TGA-CGG-CTT-TGT-3′ (R) [7]. The presence of these genes in the *E. coli* strains was determined by fragment sizes of 163, 368, 275, and 111 bp for STa, STb, LT, and EAST1, respectively. Polymerase chain reaction products were separated through 2.0% agarose gel electrophoresis, stained with 1× GelRed (Sigma-Aldrich, USA), and visualized under a UV transilluminator UVP (Nippon Genetics).

The strains *E. coli* Laboratory (ECL) 6611 and ECL 7805, which were kindly provided by the Reference Laboratory of the University of Montreal (Montreal, Canada), were used as positive controls for the presence of toxin genes in PCR.

## Results

### Isolation of presumptive *E. coli* strains and evaluation of their hemolytic ability

Fifty-two presumptive *E. coli* strains were isolated from rectal swabs collected from the selected farms. Only six (11.5%) presumptive *E. coli* strains were positive for β-hemolysis. These hemolytic strains were isolated from an intensive swine farm (village of El Bolo, Palmira, Valle del Cauca, Colombia) and identified as 1:17, 1:18, 1:19, 2:3, 2:4, and 2:5 for further analysis.

### Molecular identification of the pathotype of *E. coli*

16S ribosomal gene fragments of 1341 bp from six presumptive *E. coli* strains were amplified by PCR and sequenced. The nucleotide sequences were analyzed with BLASTn, and the isolated strains had 99.33%–100% identity with *E. coli* strains.

These results confirmed that the hemolytic strains isolated from rectal swabs corresponded to *E. coli* species. The nucleotide sequences were submitted to the GenBank database (https://www.ncbi.nlm.nih.gov/genbank/) under the accession codes OM757876 (1:19), OM757877 (2:5), OM757878 (1:17), OM757879 (2:4), OM757880 (1:18), and OM757881 (2:3).

### Evaluation of adhesion factor genes

**Figure-1:**
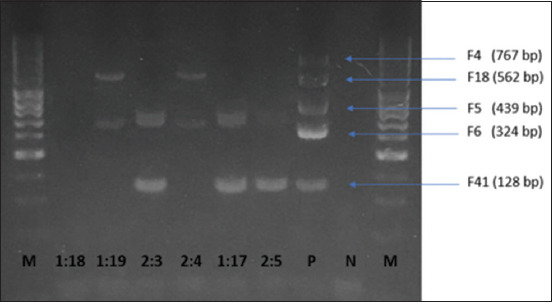
Multiplex polymerase reaction for the detection of adhesion factor genes F4, F5, F6, F18, and F41 in *Escherichia coli* strains positive for β-hemolysis. Each of the *E. coli* strain samples was identified in its corresponding lane 1:18, 1:19, 2:3, 2:4, 1:17, and 2:5. Lane P corresponds to the positive control, lane N corresponds to the negative control, and lanes M correspond to the molecular weight marker (50–1000 bp).

A gel electrophoresis image of the multiplex PCR performed to evaluate *E. coli* adhesion factors is shown in Figure-1. Adhesive fimbriae were found in five of six β-hemolytic *E. coli* isolates. OM757876 and OM757879 carried F6 and F18, and strains OM757881, OM757878, and OM757877 carried F6, and F41. Strain OM757880 was negative for the adhesion factors studied.

### Enterotoxin gene evaluation

The presence of toxin genes in the reference control (ECL 6611 and ECL 7805) strains was verified by PCR. The controls presented bands for the toxin genes STa (163 bp), STb (368 bp), LT (275 bp), and EAST1 (111 bp). The six hemolytic strains isolated in this study were negative for all four toxin genes (data not shown).

### Antibiotic resistance of hemolytic *E. coli* strains

The antimicrobial susceptibility of *E. coli* isolates to nine antimicrobial agents is shown in Table-2. Presumptive *E. coli* strains negative for hemolysis (46 strains) were more susceptible to FOS (28 strains) and AK (13 strains). In contrast, none of these strains was sensitive to AMP, whereas one strain each had low sensitivity to OD and FFC. In the non-hemolytic group, four strains were resistant to all nine antibiotics, and 11 strains were sensitive to only one antibiotic.

**Table-2:** Antibiotic resistance profiles of 52 presumptive *E. coli* strains isolated from rectal swabs in piglets.

Strains	AK	AMP	CIP	DO	ENR	FFC	FOS	CN	NOR	APR	EFT
1.1	S	R	R	R	R	R	R	R	R		
1.3	R	R	R	R	R	R	R	R	R		
1.4	S	R	R	R	R	R	I	R	R		
1.5	R	R	R	R	R	R	S	R	R		
1.7	S	R	R	R	R	R	R	R	R		
1.8	R	R	R	R	R	R	S	R	R		
1.9	R	R	R	R	R	R	R	R	R		
1.10	S	R	S	I	I	R	S	R	S		
1.11	R	R	R	R	R	R	S	R	R		
1.12	R	R	R	R	R	R	S	R	R		
1.13	R	R	R	R	R	R	R	R	R		
1.14	S	R	R	R	R	R	S	R	R		
1.15	R	R	R	I	R	R	S	I	R		
1.16	R	R	S	R	I	R	S	R	R		
*1.17	R	R	R	R	R	R	S	S	I	R	R
*1.18	R	R	R	R	R	R	S	S	R	R	R
*1.19	S	R	S	R	R	R	S	S	S	R	R
2.2	R	R	R	R	R	R	S	R	R		
*2.3	R	R	S	R	R	R	S	S	R	R	R
*2.4	R	R	R	R	R	R	S	S	I	R	R
*2.5	R	R	R	R	I	R	S	S	S	R	R
2.6	R	R	R	R	R	R	S	I	R		
2.7	R	R	R	R	R	R	S	R	R		
2.8	R	R	S	R	S	R	R	R	S		
2.9	R	R	R	R	R	R	S	I	R		
2.10	R	R	R	R	R	R	S	R	R		
2.12	R	R	R	R	R	R	S	I	R		
2.13	S	R	R	R	R	R	S	R	R		
2.14	R	R	S	R	I	R	S	R	R		
2.15	R	R	R	R	R	R	S	R	R		
2.16	S	R	R	R	R	R	R	S	R		
2.17	R	R	R	R	R	R	R	I	R		
2.18	S	R	R	R	R	R	S	S	R		
2.19	R	R	R	R	R	R	S	I	R		
2.20	R	R	R	R	R	R	R	R	R		
3.1	S	R	R	I	R	R	S	S	R		
3.3	S	R	R	R	R	R	S	R	R		
3.4	R	R	R	I	R	R	S	R	R		
3.5	R	I	I	S	R	S	S	S	R		
3.6	R	R	S	R	S	R	S	S	S		
3.7	R	R	I	R	I	R	S	I	I		
3.8	R	R	S	R	S	R	S	S	S		
3.9	R	I	S	R	I	R	S	I	R		
3.10	R	I	I	R	I	R	S	I	I		
4.2	R	R	I	R	I	R	R	S	R		
4.4	S	R	R	R	R	R	R	S	R		
4.6	S	R	S	I	I	R	R	R	S		
4.10	R	R	S	I	I	R	R	S	I		
4.13	S	I	I	I	R	I	R	S	R		
4.15	R	R	I	R	I	R	R	R	S		
4.17	R	R	I	I	I	R	R	S	R		
4.19	R	R	S	R	I	R	R	R	I		

* Corresponds to hemolytic *E. coli* strains. *S=Sensitive, I=Intermediate, R=Resistant, AK=Amikacina, AMP=Ampicilina, CIP=Ciprofloxacina, DO=Doxiciclina, ENR=Enrofloxacina, FFC=Florfenicol, FOS=Fosfomicina, CN=Gentamicina, NOR=Norfloxacina, APR=Apramycin, EFT=Ceftiofur, *E. coli*=*Escherichia coli*

Six β-hemolytic *E. coli* isolates were tested for antimicrobial susceptibility to 11 different antibiotics (Table-2). All *E. coli* strains tested were resistant to AMP, FFC, DO, EFT, APR, and ENR. Only strain OM757877 exhibited intermediate susceptibility to ENR. Strain OM757880 was the most resistant strain, displaying resistance to 8 of 11 antibiotics tested. The strain with the lowest antibiotic resistance was strain OM757876, which was resistant to six of 11 antibiotics. All tested strains were susceptible to CN and FOS.

## Discussion

A correct early diagnosis and understanding of which herd-specific pathotypes of *E coli* are present during a diarrhea outbreak are of high clinical relevance [26]. In this work, β-hemolytic *E. coli* strains with adhesion factors and resistance to antibiotics were isolated from the rectal swabs of piglets with evidence of diarrhea. To the best of our knowledge, this is the first report of virulence factors of *E. coli* strains associated with diarrhea in piglets in Columbia. In contrast, several studies describing the presence of colibacillosis in swine have been published in other Latin American countries such as Mexico [12], Brazil [22], and Argentina [27].

In the present study, the evaluation of hemolytic activity was used as an initial screening test to identify possible virulence factors present in *E. coli* strains isolated from piglets with diarrhea. Based on this characteristic, we proceeded to detect adhesion factors, enterotoxins, and antibiotic sensitivity. Fifty-two *E. coli* strains were isolated from the rectal swabs of piglets with diarrhea. However, only six strains showed hemolytic activity. Genes for adhesive fimbriae were found in five of six hemolytic strains. Nevertheless, toxin genes were not detected in these strains, representing an interesting finding, whereas adhesion factors and resistance to as many as eight antibiotics were detected in five of these strains.

In piglets, diarrhea might be associated with the proliferation of one or more strains of β-hemolytic ETEC, particularly those that express fimbrial genes, in the small intestine. In one study, Toledo *et al*. [12] found that only 8.7% of 953 *E. coli* strains isolated from piglets with diarrhea exhibited β-hemolysis. In contrast, Sato *et al*. [22] observed that 47.4% of 287 *E. coli* isolates displayed β-hemolysis.

Different studies have assessed the association of the hemolytic activity of *E. coli* strains with their pathogenic virulence [28]. A significant association between the presence of virulence factors (F4, F18, Sta, and STx2) and β-hemolytic activity has been described [22, 29]. Although hemolysis in *E. coli* is not an absolute indication of pathogenicity in pigs, a test for hemolysis can be a useful indicator of the pathogenicity of *E. coli* causing disease in piglets or at least an association with the presence of fimbriae genes, as observed in the present study, in which it was used as a preliminary screening test [29].

This study examined the presence of genes for adhesins and enterotoxins representing the virulence factors and their associations in *E. coli* isolated from piglets with diarrhea. Nevertheless, we must consider that the samples collected in this study were limited to six farms in a single pig-producing region of our country, and *E. coli* was isolated from 52 samples. However, among them, only six isolates were hemolytic strains. Of these strains, five carried virulence-associated genes. Two strains carried the adhesion factors F6 and F18, and three strains carried the fimbriae F6 and F41. F6 and F41 have been associated with diarrhea in suckling piglets, whereas F18 has been associated with diarrhea and edema disease in weaned piglets [2, 30]. The prevalence of virulence factors of pathogenic *E. coli* strains can vary by region and country [12, 31], which might explain the low number of virulent isolates detected in our study.

The β-hemolytic *E. coli* strains OM757878, OM757876, OM757881, OM757879, and OM757877 did not carry toxin genes, suggesting that these strains have pathogenic ability because they carry combinations of β-hemolytic and adhesion factors. The combination of virulence factors might determine the pathogenicity of *E. coli* strains, as previously reported by Toledo *et al*. [12], Kim *et al*. [29], Renzhammer *et al*. [31], and Pitout [32]. Conversely, diarrhea in piglets in which no pathogenic *E. coli* strains were identified is explained by the presence of one or more unidentified etiologic agents, such as *Cystoisospora suis*, *Clostridium perfringens*, and rotavirus (A and C).

Antimicrobial susceptibility testing showed that hemolytic strains carrying adhesion factors were resistant to some antibiotics. Consequently, diarrheal disease caused by these resistant *E. coli* strains might be difficult to treat in piglets on affected farms.

Thus, the possibility of the transmission of antibiotic resistance genes from animals to humans is a serious public health concern, as previously reported by Monger *et al*. [33]. Antibiotic resistance mechanisms are encoded by different genes that can be transferred between bacteria vertically (between generations) or horizontally. Horizontal transfer is the most common form, and it occurs through the transmission of genes through mobile elements of genetic material such as plasmids and transposons. Thus, resistance mechanisms are easily propagated between individuals [34, 35]. It has also been observed that the microorganisms found in production animals are important reservoirs of antibiotic resistance genes; likewise, an increase in the presentation of antibiotic resistance among pathogenic microorganisms that affect swine herds has been reported by Monger *et al*. [31] and Vila *et al*. [36]. Although there is no indication that drug resistance enhances the virulence of *E. coli* associated with diarrhea, some virulence genes might be associated with drug resistance genes [13, 37]. In this study, PCR did not determine the resistance genes carried by the examined strains and their locations in plasmid-mediated resistant genes or the bacterial chromosome.

## Conclusion

The results of this research illustrated that *E. coli* strains with pathogenic characteristics, such as hemolytic capacity, adhesion factors, and resistance to multiple antibiotics, are circulating in Valle del Cauca, Colombia. The importance of our findings is that the circulation of strains with these characteristics can become a serious public health problem because antibiotic resistance genes can be transferred to other pig farms and humans.

## Authors’ Contributions

OVP: Performed the experiments, organized the results, and participated in writing of the manuscript. KL: Advised on the molecular biology experiments and the writing of the manuscript. GAC: Advised on the selection of piglets, sampling, and methodology for virulence factor determination, and writing of the of the manuscript. JDM: Advised on the selection of piglets, sampling, and methodology for determining virulence factors, and writing of the manuscript. LS: Participated in the research idea, contributed to the interpretation of the results and the writing of the manuscript. All authors have read, reviewed, and approved the final manuscript.
